# Integrated approach for detection of SARS-CoV-2 and its variant by utilizing LAMP and ARMS-PCR

**DOI:** 10.1186/s12941-023-00665-0

**Published:** 2024-02-01

**Authors:** Maryam Nawab, Syeda Kiran Riaz, Eiman Ismail, Alfar Ahamed, Aaysha Tariq, Muhammad Faraz Arshad Malik, Naeem F. Qusty, Farkad Bantun, Petr Slama, Massab Umair, Shafiul Haque, D. Katterine Bonilla-Aldana, Alfonso J. Rodriguez-Morales

**Affiliations:** 1 Department of Biosciences, COMSATS University Islamabad, Islamabad, Pakistan; 2 Department of Molecular Biology, Shaheed Zulfiqar Ali Bhutto Medical University, Islamabad, Pakistan; 3 Molecular Diagnostic Unit, Clinical Pathology Department, PAEC General Hospital, Islamabad, Pakistan; 4 Laboratory Medicine Department, Faculty of Applied Medical Sciences, Umm Al-Qura University, PO Box 7607, Makkah, Al Abdeyah Saudi Arabia; 5 Department of Microbiology, Faculty of Medicine, Umm Al-Qura University, Makkah, Saudi Arabia; 6 Laboratory of Animal Immunology and Biotechnology, Department of Animal Morphology, Physiology and Genetics, Faculty of AgriSciences, Mendel University in Brno, Brno, 61300 Czech Republic; 7 Department of Virology, National Institute of Health (NIH), Islamabad, Pakistan; 8 Research and Scientific Studies Unit, College of Nursing and Health Sciences, Jazan University, Jazan, 45142 Saudi Arabia; 9 Gilbert and Rose-Marie Chagoury School of Medicine, Lebanese American University, Beirut, 1102 2801 Lebanon; 10 Centre of Medical and Bio-Allied Health Sciences Research, Ajman University, Ajman, 13306 United Arab Emirates; 11 Research Unit, Universidad Continental, Huancayo, 12000 Peru; 12 Master Program on Clinical Epidemiology and Biostatistics, Faculty of Health Sciences, Universidad Científica del Sur, Lima, 15046 Peru

**Keywords:** SARS-CoV-2, RT-LAMP, ORF1ab, Q57H, ARMS-PCR

## Abstract

Global impact of COVID-19 pandemic has heightened the urgency for efficient virus detection and identification of variants such as the Q57H mutation. Early and efficient detection of SARS-CoV-2 among densely populated developing countries is paramount objective. Although RT-PCR assays offer accuracy, however, dependence on expansive kits and availability of allied health resources pose an immense challenge for developing countries. In the current study, RT-LAMP based detection of SARS-Cov-2 with subsequent confirmation of Q57H variant through ARMS-PCR was performed. Among the 212 collected samples, 134 yielded positive results, while 78 tested negative using RT-LAMP. Oropharyngeal swabs of suspected individuals were collected and processed for viral RNA isolation. Isolated viral RNA was processed further by using either commercially available WarmStart Master Mix or our in house developed LAMP master mix separately. Subsequently, the end results of each specimen were evaluated by colorimetry. For LAMP assays, primers targeting three genes (ORF1ab, N and S) were designed using PrimerExplorer software. Interestingly, pooling of these three genes in single reaction tube increased sensitivity (95.5%) and specificity (93.5%) of LAMP assay. SARS-CoV-2 positive specimens were screened further for Q57H mutation using ARMS-PCR. Based on amplicon size variation, later confirmed by sequencing, our data showed 18.5% samples positive for Q57H mutation. Hence, these findings strongly advocate use of RT-LAMP-based assay for SARS-CoV-2 screening within suspected general population. Furthermore, ARMS-PCR also provides an efficient mean to detect prevalent mutations against SARS-Cov-2.

## Introduction

*Severe acute respiratory syndrome-related coronavirus* (SARS-CoV-2), arising from a novel human *Betacoronavirus*, was initially identified in 2019 at the seafood market in Wuhan, China. World Health Organization (WHO) declared it a global pandemic due to its widespread transmission and infectious nature [1]. Since these viruses can affect a wide range of organisms, including mammalian and avian species, they pose a serious threat to human health. Viral strains that are more virulent or resistant to treatment interventions than the original ones may arise from the fast evolution of their genomic RNA through recombination [2]. Being 5th largest population, Pakistan also encountered an immense challenge of disease diagnosis and treatment during the COVID-19 outbreak. The situation was aggravated by sharing geographical borders with the region’s two most adversely affected countries (China & Iraq) [3]. According to NCOC’s recent statistics, ~ 1.5 million confirmed cases and 30,364 fatalities have been reported in Pakistan (World Health Organization).

Coronavirus is an enveloped single-stranded RNA virus with a diameter range of 80–220 nm. Coronaviruses contain five critical genes, including four structural genes, namely Nucleocapsid (N), Enveloped (E), Spike (S) and Membrane (M), along with viral polymerase (RNA dependent RNA polymerase (RdRp) [4]. Coronavirus comprises 29,811 nucleotides with 14 Open Reading Frames (ORFs) responsible for encoding 27 proteins. At the 5’-terminus, there are 16 non-structural proteins involved in immune evasion [5]. There are 11 accessory proteins named ORF3a, ORF3b, ORF3c, ORF3d, ORF6, ORF7a, ORF7b, ORF8, ORF9b, ORF9c, and ORF10. The functioning of these accessory proteins is yet to be deciphered in detail. Most mutations observed among the accessory proteins have been reported among variants of concern affecting disease severity [6].

Significant functions of ORF3a include pro-inflammatory cytokine and chemokine production, channel formation, facilitating viral entry, and release from the host cell. Conversion of one nucleotide at a specific position from guanine to thymine (25,563 G > T) induces the replacement of glutamine (Q) to histidine (H) at 57 positions (Q57H). All continents have shown evidence of the Q57H mutation, although Asia has seen the highest incidence. Q57H amino acid alteration in SARS-CoV-2 Orf3a causes a premature stop codon in the reading frame for Orf3b, accounting for 23.82% of genomes studied [7]. In standard Orf3a, the Q57 position was not crucial in protein-binding interfaces, but in Orf3a–S and Orf3a–Orf8 complexes, the protein change Q57H created a hot point. SARS-CoV-2 mutations may damage drug-targeting regions by altering the protein-binding interface, leading to a failure of the cure [8]. SARS-CoV-2 pathogenicity, infectivity, ion channel activity, and viral release are all linked to Q57H in ORF3a [9].

Data regarding Q57H true penetrance primarily relies on high throughput genomic sequencing. Earlier in a study, the detection of 6 clades of SARS-CoV-2 was developed by using the Amplification Refractory Mutation System Polymerase Chain Reaction (ARMS-PCR) assay [10].

Identifying SARS-CoV-2 among suspected individuals during the global outbreak is a challenge. Generally, the Real-Time Reverse Transcription-Polymerase Chain Reaction (RT-PCR) is considered the most reliable and sensitive way to identify SARS-CoV-2 infection [11]. A rapid and easy-to-use testing equipment RT-LAMP that is a simple and affordable approach is desperately needed. A kit like this would make it possible to identify virus-infected persons immediately, allowing for a quick quarantine to stop the infection’s spread. It is perfect for usage at airports, train stations, hospitals, including regional and rural medical centers, due to its portability and straight-forward result interpretation [12].

Rapid nucleic acid amplification is made possible by a technique called loop-mediated isothermal amplification (LAMP). It uses a DNA polymerase with chain displacement activity together with 4–6 distinctive primers. The strand displacement ability of the specialized polymerase removes the need for heat-induced denaturation of DNA. Compared to a thermal cycler, the device is more affordable and at a single constant temperature of 65 °C [13]. Interestingly, the Food and Drug Administration (FDA) has also approved RT-LAMP-based identification of SARS-CoV-2 [14]. RT-LAMP test may also be used in general laboratory settings as it is approved under the emergency utilization assay category [15].

The amplification of viral genetic components occurs at a stable temperature; therefore, RT-LAMP diagnostic tests may be performed everywhere with essential equipment like a heat block [16]. Using pH-based colorimetric assays is the most efficient method for diagnosing Coronavirus [17]. When complexometric indicators and pH-sensitive dyes are added, LAMP colorimetric approaches will detect turbidity based on the accumulation of magnesium pyrophosphate or color changes [16]. When amplification occurs, the reaction mixture’s pH falls as hydrogen ions accumulate. Thus, a positive result may be confirmed by visually seeing if the reaction solution turns pink to yellow [18].

In this study, a one-tube colorimetric In-house Reverse-transcription loop-mediated isothermal amplification (RT-LAMP) assay is used for the visual detection of SARS-CoV-2 RNA. The aim of the current study is the screening of SARS-CoV-2 genes by using RT-LAMP and pooling of ORF1ab, N and S genes for assessment of increase in accuracy of the kit. Moreover, identification of ORF3a variant (Q57H) via ARMS-PCR has also been performed to provide a reliable approach for early detection of VOC.

## Methodology

### Ethical approval

The study was approved by ethical and biosafety committees of COMSATS University Islamabad and the National Institute of Health (NIH), Pakistan. Oropharyngeal swabs of suspected individuals were collected with signed consent and standardized biosafety protocols were followed at NIH for the needful collection of specimens.

### Isolation of RNA and cDNA synthesis

Swabs were processed for RNA isolation using Invitrogen Pure Link RNA Mini Kit as per manufacturer guidelines. RNA of the suspected specimen was initially tested using RT-PCR for the presence of the virus. Later, the same specimen was used to develop a LAMP-based SARS-CoV-2 assay.

### Designing of LAMP-based primers for ORF1ab, N and S gene

LAMP-based primer sets targeting ORF1ab, N and S gene (patent No. 157/2022) were designed for SARS-CoV-2 by using Primer V5 software. Primer-BLAST analysis of the designed primers was also performed to check for non-specific amplification.

### Reverse transcriptase- loop-mediated isothermal amplification (RT-LAMP)

A WarmStart Colorimetric RT-LAMP 2X Master Mix (RNA) was used to perform RT-LAMP-based detection of SARS-CoV-2. The reaction mixture composed of Master Mix (1.5 µl), primer (2.5 µl), water (8 µl) and RNA sample (2 µl) was carried out at 60ºC for 40 min. The product obtained after amplification was visually examined by its color, where yellow represents the presence of virus and pink shows its absence.

### Optimization of in-house LAMP assay

Several reactions were performed on different temperature ranges (60 ºC − 65 ºC) to optimize the LAMP assay, where 61ºC was found to be the best optimum temperature. Multiple reactions were carried out to optimize the quantity of various reagents, particularly BSM polymerase and RT enzyme, within the In-House master mix formulation. Finally, the reaction mixture comprised of 10X buffer (1 µl), MgCl_2_ (1.6 µl), dNTPs (1.4 µl), water (1 µl), primer pairs (1 µl), BSM polymerase (0.5 µl), RT enzyme (0.5 µl) and RNA sample (3 µl) was optimized. Based on extensive experimentation, incubation at 61ºC for 1 h was a more appropriate condition for LAMP assay. The presence of the virus was confirmed by the gel electrophoresis method.

### In-house colorimetric RT-LAMP assay

LAMP reaction mix consisted of 0.25 µl of 10x buffer, 1.6 µl of Mgcl_2_, 1.4 µl of dNTPs, 1 µl of dye, 1 µl of primer, 0.5 µl of BSM polymerase, 0.5 µl of RT enzymes, 0.75 µl of DEPC water, and 3 µl of RNA sample which was heated for 58 min at 61ºC. The pH of the reaction solution decreases as hydrogen ions accumulate during the amplification reaction. The result was confirmed visually by examining whether the reaction solution changes color or not.

### Confirmation by real time-PCR (RT-PCR)

cDNA synthesized was processed further to assess the presence of SARS-CoV-2 genes (ORF1a, N and S). Detection of SARS-CoV-2 was done by utilizing a commercially available kit (BGI Genomics, Shenzhen, China) as per manufacturer instructions [19].

### Designing and optimisation of amplification refractory mutation system (ARMS-PCR) for analysis of VOC

The genomic sequence of SARS-CoV-2 (NC 045512.2) was retrieved from NCBI. Four independent primers were designed to target the region of ORF3a in general and 25563G > T (Q57H) in particular. Outer forward primer CAAATTTGATGAAGACGACTCTGAGCCA and outer reverse primer AGATAGAGAGAAGGGGCTTCAAGGCCAG yield a product size of 393 bp. Using internal forward primer TGGCGTTGCACTTCTTGCTGTTTTTTAT and internal reverse primer GAGGGTTATGATTTTGGAAGCGCCAC, two different amplicon product sizes were expected. Amplicons of 265 bp and 182 bp were expected for the wild-type and mutant variants, respectively. Reagents used for this reaction include 5X master mix (2 -20 µl), 10mM outer forward primer and 10mM outer reverse primer (1 − 10 µl), 10mM inner forward and inner reverse primer (0.5 − 5 µl), distilled water (3 − 30 µl) and cDNA (2 -12 µl) (Revert Aid First Strand cDNA Synthesis Kit, Thermo Scientific Fisher, USA). Reaction conditions include 95 °C for 5 min followed by 35 cycles of 94 °C for 30s, 58 °C for 1 min, 72 °C for 1 min with a final extension at 72 °C for 10 min.

### Post amplification horizontal gel electrophoresis method and sequencing

Amplicons synthesized were run on 2% agarose gel along with a 100 bp ladder. Specimens identified as positive for Q57H mutations were sequenced using the same primers for Sanger sequencing.

### pH-based colorimetric detection

A pH-sensitive dye (Cresol red) and a combination of dyes (Phenol red + Azure II) were selected based on the pH range of RT-LAMP and the level of sensitivity and accuracy. These dyes showed a convenient color change for the point of care (POC) detection. The cresol red dye can change from pink to yellow when the reaction pH changes from basic to acidic. As well as the combined dye of phenol red and azure II can change the color from purple/blue to slightly green when the reaction of pH alters from basic to acidic.

### Statistical analysis

Statistical analysis of the data was performed with IBM SPSS Software (USA). Data was analyzed by applying different statistical tests including T test, ANOVA and ROC Curve. Area Under Curve (AUC) with 0.9-1.0 was considered as statistically significant. Sensitivity and Specificity was also evaluated for RT-LAMP assay using the same software.

## Results

### Clinical parameters of the cohort

A total of 212 nasal and oropharyngeal samples were collected from NIH, Islamabad, Pakistan. These samples were processed and results were interpreted using RT-LAMP, RT-PCR and ARMS-PCR assays. All three genes (ORF1ab, Nucleotide (N) and Spike (S) were identified independently and in combination too. To ensure the accuracy of the results, each sample was examined through RT-LAMP, and RT-PCR was used to confirm the results of these samples. Sensitivity of RT-LAMP screening was found to be 95.5% and specificity was 93.6% for the detection of SARS-CoV-2 virus in Table 1.

**Table 1:** Tabular presentation of the results

Total samples (212)	134 Samples		78 Samples	
	Positive	Percentage	Negative	Percentage
RT-LAMP	128	95.5%	73	93.6%
RT-PCR	134	100%	78	100%

### ORF1ab, N and S results confirmation

**Fig. 1:**
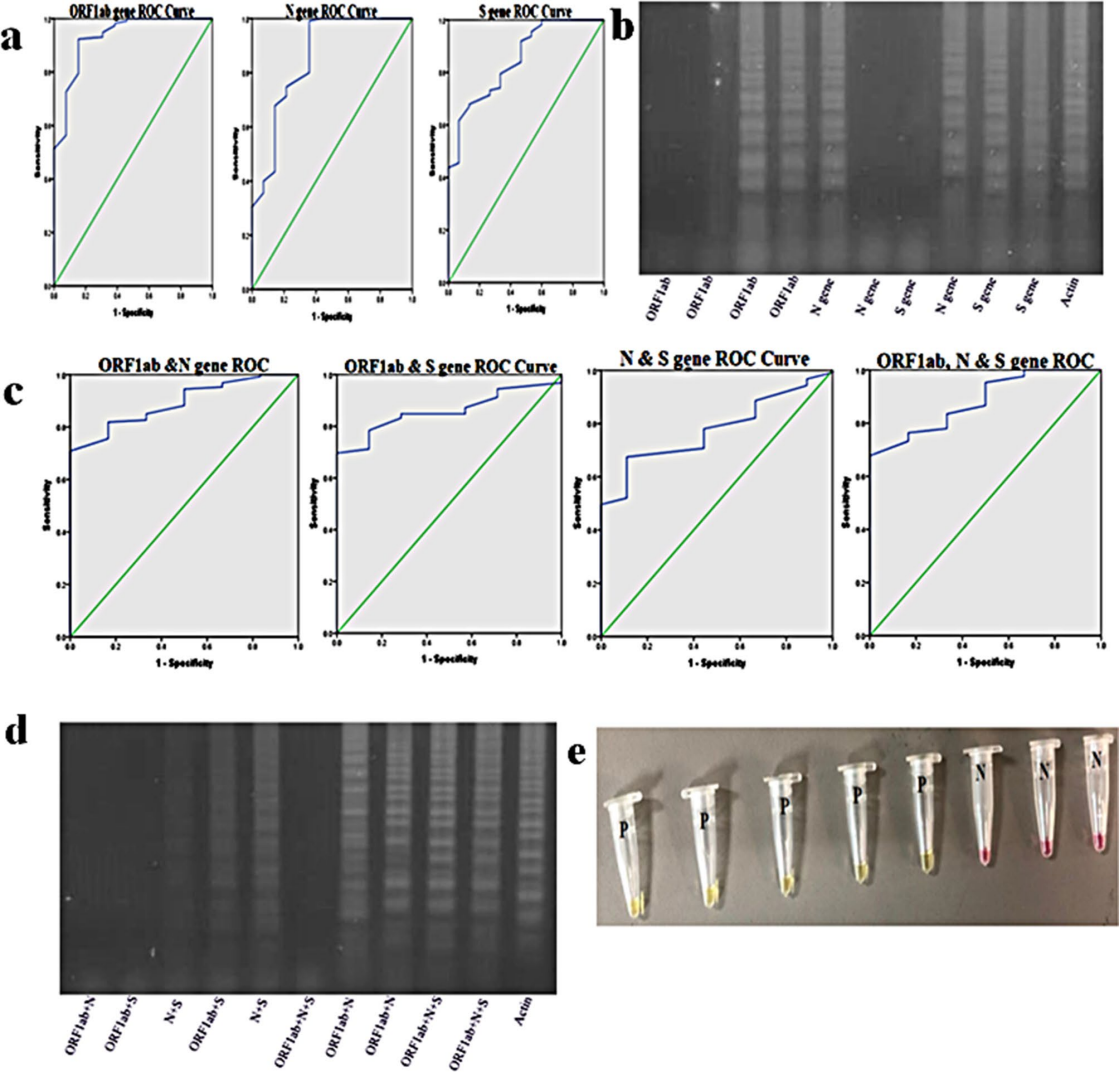
Sensitivity and specificity of SARS-CoV-2 genes. (**a**) Representative Image of SARS-CoV-2 genes (ORF1ab, N and S): (**b**) Agarose gel electrophoresis showing individual amplification of each gene: (**c**) ROC curve analysis of pooling of different genes: (**d**) Banding pattern upon pooling of these genes shown on agarose gel electrophoresis: (**e**) Colorimetric results of ORF1ab, N, S and actin gene via WarmStart Master Mix for LAMP where the yellow color indicate the presence of the virus while the pink color shows the absence of the virus (SARS-CoV-2)

Both colorimetric (WarmStart Master Mix) and gel electrophoresis (RT-LAMP) techniques were used to confirm SARS-CoV-2 in RT-LAMP assay. A cluster of 4 genes were used, including ORF1ab, N and S (representative of SARS-CoV-2) as well as Actin (internal control). Detailed analysis of the ORF1ab gene showed 87.3% sensitivity and 85.8% specificity with RT-LAMP assay along with the predictive value, accuracy and confidence intervals as mentioned in Table 2. The graphic representation and gel electrophoresis are also shown in Fig. 1a, b.

**Table 2:** Statistical analysis of ORF1ab, N and S gene individually

							Asymptotic 95% Confidence Interval	
Genes	Sensitivity	Specificity	PPV	NPV	Accuracy	Area	Lower Bound	Upper Bound
ORF1ab	87.3%	85.8%	91.41%	79.76%	86.79%	0.931	0.857	1.000
N	85.8%	84.62%	90.55%	77.65%	85.38%	0.856	0.736	0.976
S	83.58%	80.77%	88.19%	74.12%	82.55%	0.850	0.757	0.943

The statistical analysis was performed on SPSS software for each gene, where sensitivity, specificity, PPV (Positive Predictive Value), NPV (Negative Predictive Value), Accuracy, Area and Confidence Interval were calculated.

### Sensitivity of the assay increased by pooling ORF1ab, N and S genes

Various combinations of ORF1ab, N and S pooling in a single tube were also tested to monitor the sensitivity and specificity of the assay. These genes were pooled and then 1 µl was added to the in-house RT-LAMP Master mix. Interestingly, the pooling of ORF1ab, N and S genes showed high sensitivity (95.5%) and specificity (93.6%) compared to individual genes, as shown in Table 3. ROC Curve analysis of Pooled genes and its gel image is shown in Fig. 1c, d.

**Table 3:** Statistical analysis of pooling-based amplification

							Asymptotic 95% Confidence Interval	
Pooling of Genes	Sensitivity	Specificity	PPV	NPV	Accuracy	Area	Lower Bound	Upper Bound
ORF1ab & N	94.78%	92.31%	95.49%	91.14%	93.87%	0.892	0.806	0.977
ORF1ab & S	93.28%	91.03%	94.70%	88.75%	92.45%	0.859	0.780	0.938
N & S	91.79%	88.46%	93.18%	86.25%	90.57%	0.777	0.670	0.884
ORF1ab, N & S	95.52%	93.59%	96.24%	92.41%	94.81%	0.882	0.782	0.982

Data showed that the pooling of SARS-CoV-2 genes shows an increase in the ratio of sensitivity and specificity as compared to the single gene. A representative image of the ROC Curve analysis of each gene via SPSS, gel electrophoresis and warm start Master Mix is also shown in Fig. 1.

### In-house colorimetric assay of different genes

**Fig. 2:**
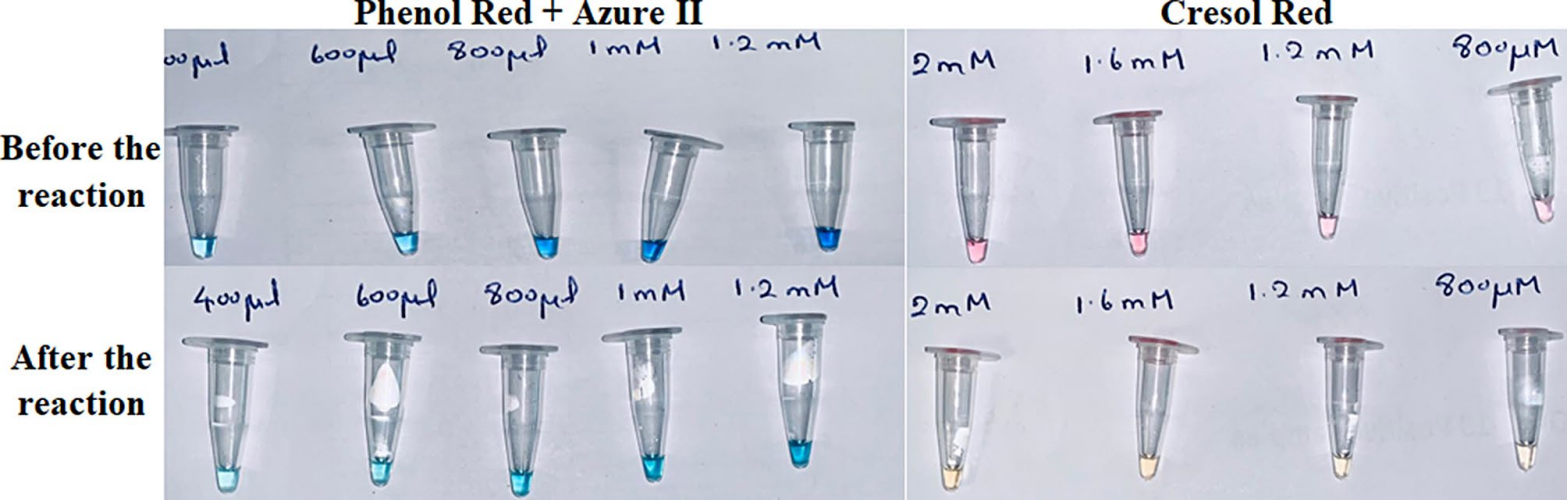
In-house colorimetric Assay of SARS-CoV-2 genes. Phenol red and Azure II dye combine to give a blue color, while a modest green coloring that results from the RT-LAMP Assay indicates the presence of a virus. Whereas, the cresol red, which was pink before the reaction and became yellow, confirms the existence of the SARS-CoV-2 gene

Within the In-house RT-LAMP master mix, a dye indicates evident color change, which ensures that the RT-LAMP products can be visualized with the naked eye for detection at POC. This in-house colorimetric LAMP assay was performed on all SARS-CoV-2 genes (ORF1ab, N and S). Many reactions were carried out at various concentrations throughout the procedure to optimize the dye concentration and reagent volume. The yellow cresol red in the reaction shows the presence of the virus, and the slight green color of the combined dye identifies the positive sample, as shown in Fig. 2.

A total of 134 samples were analyzed for each gene using ANOVA tests as shown in Table 4.

**Table 4:** Analysis of SARS-CoV-2 genes for colorimetric assay

	ORF1ab Gene	N Gene	S Gene	ORF1ab + N pooling	ORF1ab + S pooling	N + S pooling	ORF1ab + N + S
P value	< 0.0001						
Mean	0.1269	0.1418	0.1642	0.05224	0.06716	0.08955	0.04478
Std. Deviation	0.3341	0.3501	0.3718	0.2233	0.2512	0.2866	0.2076
Std. Error of Mean	0.02886	0.03025	0.03212	0.01929	0.02170	0.02476	0.01793

### ARMS-PCR for detection of VOC

Among the 300 swabs samples, 135 tested positive for the presence of SARS-CoV-2, with a product size of 393 bp. Out of these positive samples, 110 showed typical traits of the wild-type strain (265 bp and 182 bp fragments). The remaining 25 samples, meanwhile, showed that a variation was present.

### Confirmation of variants by sequencing

**Fig. 3:**
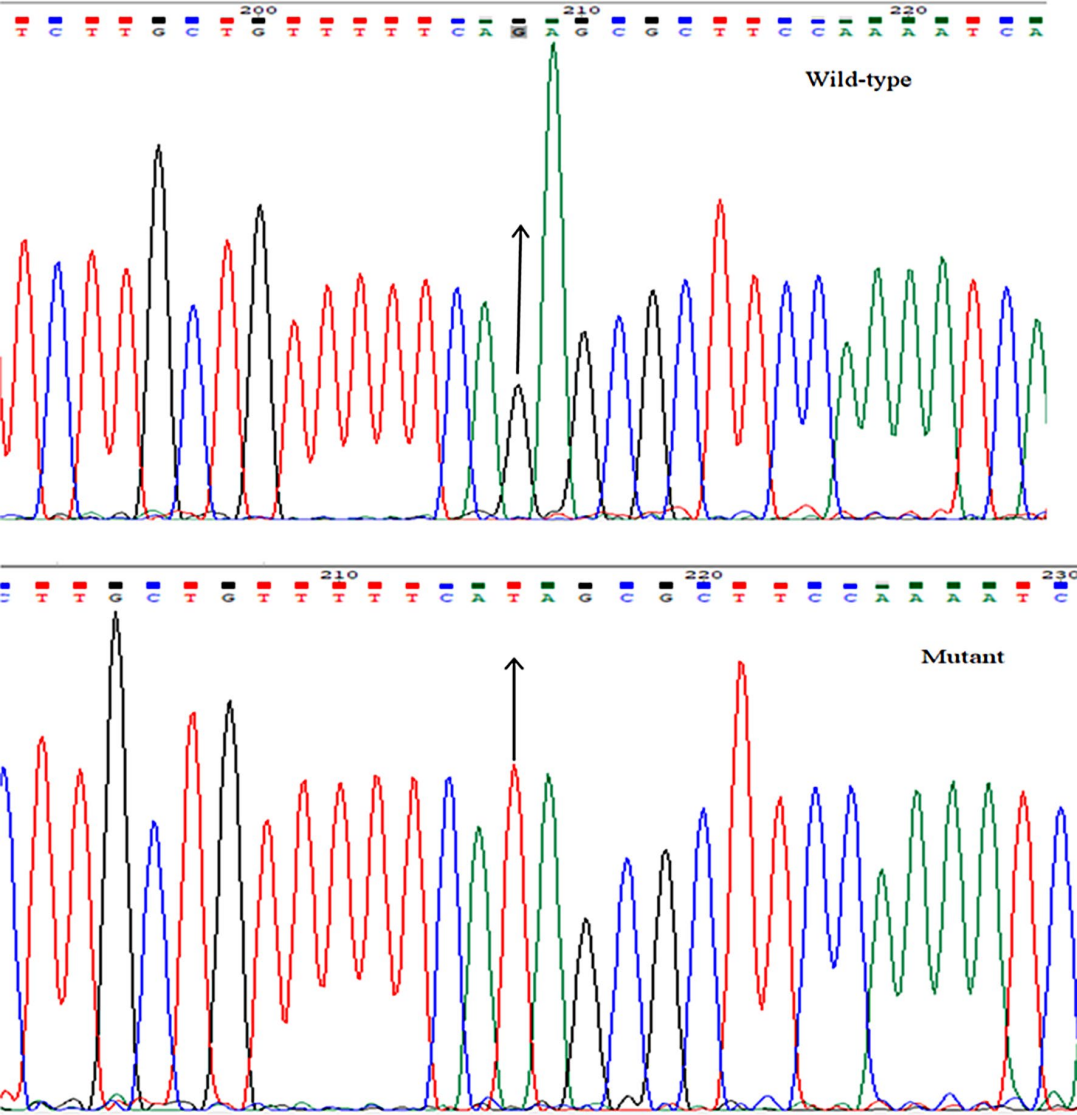
Representative image of sequencing results showing single nucleotide mutation at 57 positions of orf3a protein (With the conversion of G into T)

Both wild-type and mutant forms were also confirmed by sequencing, as shown in Fig. 3. It shows G > T conversion that corresponds to the change of glutamine amino acid (Q) with histidine (H) at position 57, as shown in Fig. 3.

## Discussion

According to the Center for Disease Control and Prevention (CDC) recommendation, the detection of SARS-CoV-2 is mainly dependent on RT-PCR assay (Hueda-Zavaleta et al., 2022). However, an exhaustive list of real-time PCR challenges, including sample collection errors, inadequate material volume, and interfering substances, also compromises the COVID-19 detection reliance solely on RT-PCR assay [20]. Based on limited viral load, the chances of detecting false negative results are high during the early and late phases of the disease. False results from RT-PCR assays were also reported among commercially available SARS-CoV-2 detection kits. One of the plausible reasons for these results is generally attributed to virus sequence variation, especially on the target regions of ORF1ab and N gene [21]. In another study, the sensitivity of 6 different diagnostic kits indicated a wide range of false negative (2-39%) results for SARS-CoV-2 among 98 specimens [22]. These variations never undermine the importance of real-time PCR in diagnostics. However, the challenges of acquiring expensive instruments, training human resources, and addressing troubleshooting are immense for a developing country.

RT-LAMP-based screening assay was developed to utilize existing laboratory instrumentations and limited dependency on trained human resources, especially in global emergencies [23]. The present study developed an In-House RT-LAMP assay to detect SARS-CoV-2 using existing laboratory settings easily.

Comparative analysis showed that the assay had accurately identified 128 out of 134 COVID-19 positive cases. The current study achieved 95.5% overall sensitivity with 93.6% specificity compared to RT-PCR. These findings were more promising in contrast to prior research, where sensitivity (87.5%) and specificity (100%) were markedly low against the N gene at the Massachusetts General Hospital [24].

Based on individual gene screening, sensitivity values of ORF1ab, N and S genes observed were 87.3%, 85.8% and 83.5% respectively. The specificity values of ORF1ab, N and S gene for SARS-CoV-2 were 85.8%, 84.6% and 80.7% respectively. In another study, LAMP-based screening of SARS-CoV-2 by targeting the S region yielded 88.9% sensitivity and 99% specificity [25]. The reason for variable sensitivity and specificity values indicates the influence of several factors, including sample numbers, specimen quality, primer sets, and the presence of variants of concerns observed in the subsequent wave of COVID-19. To address this challenge, the pooling of these genes in one reaction vial was also done with an evidential increase in sensitivity and specificity.

With the availability of increasingly sensitive primer sets, the RT-LAMP test has a lot of potential applications. Scalable testing may be possible with the RT-LAMP assay and LAMP-sequencing, which would be challenging with traditional RT-qPCR-based diagnostics [26]. The most frequent disadvantage of this method is the binding primer secondary structures, which causes inaccurate amplification in negative samples and could result in a false-positive diagnosis. Primers must be carefully designed to prevent the formation of thesestructures. Additionally, it was shown that omitting the RNA extraction stage can result in incorrect diagnosis as it alters the pH of the solution, particularly in oropharyngeal specimen. The RNA extraction step is also essential to ensure the accuracy of the colorimetric RT-LAMP results [27].

ARMS-PCR is among one of the most widely used assays which have been designed for detecting known SNP genotypes. Based on fast processing time, its utilization in screening huge biological samples during an outbreak is highly recommended. The ARMS–PCR process is a straightforward and inexpensive way to genotype single-nucleotide polymorphisms (SNPs) [28]. It provides quick and easy identification at a low cost, and it just requires a minimal degree of skill and apparatus [29]. A single PCR followed by simple gel electrophoresis [30].

ORF3a protein of SARS-CoV-2 significantly contributes to virulence, infectivity, ion channel formation, and virus release [31]. A constriction of viroporin influenced by Q57H mutation significantly alters the entry of Ca2+, Na+, and K + ions across the membrane-based core factors, inducing positive charge repulsion and smaller pore size. Reduced Ca2 + presence inside the cell represses caspase-dependent host cell apoptosis, providing enough opportunity to increase viral proliferation inside the host cell [32]. Q57H conversion leads to premature truncation of ORF3b [33]. Other mutations in ORF3a protein are considered based on a succeeding time scale that was found to have 2nd level mutation along with Q57H. The fourth wave began in early November 2020 and was caused by a newly introduced GISAID clade GH SARS-CoV-2. Moreover, ORF3a-Q57H SARS-CoV-2 variant was found to be the VOC in the fourth epidemic wave of COVID-19. As a result, ORF3a could became a promising therapeutic target.

Here, the ARMS-PCR-based detection method provides an alternative robust approach for Q57H mutation detection without sequencing. Data also showed the specific binding for designed primers to the target site. So, they can be used to screen a large population for that mutation to discover the dissemination and infection potential of COVID-19. Hence, identifying variants like Q57H will help devise appropriate diagnostic kits and therapeutic strategies for COVID-19 affected patients in the upcoming waves.

## Conclusion

In conclusion, our research supports the efficacy of three SARS-CoV-2 screening techniques: RT-LAMP with gel electrophoresis, RT-LAMP with colorimetry, and ARMS-PCR for Q57H mutation. These affordable choices are appropriate for different lab situations. Rapid preliminary detection is provided by RT-LAMP, especially in contexts with limited resources. ARMS-PCR provides mutation-specific insights, such Q57H, giving a deeper understanding of the genomic changes in the virus. In the fight against pandemics, these screening techniques support the medical and research communities by facilitating early detection and well-informed decision-making.

## Data Availability

The study benefited from the availability of in silico datasets, which greatly enriched our research analysis. It is not applicable in this context.
